# Antibody responses to V2 loop are induced by CRF01_A E and not Clade B envelopes

**DOI:** 10.1186/1742-4690-9-S2-P100

**Published:** 2012-09-13

**Authors:** N Karasavvas, C Karnasuta, S Madnote, D Arworn, F Sinangil, S Rerks-Ngarm, RJ O'Connell, S Nitayaphan, V Ngauy, JH Kim, NL Michael, MS de Souza

**Affiliations:** 1 AFRIMS, Bangkok, Thailand; 2 Global Solutions for Infectious Diseases, South San Francisco, CA, USA

## Background

The RV144 vaccine trial of canarypox vCP1521 (ALVAC-HIV) prime and bivalent HIV-1 envelop gp120 protein subtype B/CRF01_AE boost (AIDSVAX B/E) demonstrated a significant effect in preventing HIV-1 infection. A case-control analysis suggested that variable loops 1 and 2 (V2) of gp120 may have contributed to protection against HIV-1 acquisition. Two other vaccine trials using gp120 only– VAX003 (AIDSVAX B/E) and VAX004 (AIDSVAX B/B) failed to show protection.

## Methods

Binding antibody responses induced by the RV144, VAX003 and VAX004 vaccine regimens were compared using ELISA. Recombinant gp120 envelope proteins MN (subtype B), 92TH023 (CRF01_AE), A244 (CRF01_AE) and cyclic V2 peptides were used as capture antigens.

## Results

After two protein injections, VAX004 had the highest geometric mean titers (GMT) against MN (25,600), VAX003 against A244 (21,378) and RV144 against 92TH023 (6,263). Antibody responses against V2 (CRF01_AE) were detected in plasma samples from RV144 and VAX003 with GMTs of 972 and 1100, respectively. However, VAX004 failed to generate antibodies against CRF01_AE V2. None of the three vaccines generated antibodies against MN V2 after two protein immunizations.

Compared to VAX004, VAX003 had higher antibody responses against all three recombinant proteins: 2-fold (MN), 4-fold (A244) and 4-fold (92TH023) when two additional protein injections were administered. Two additional protein inoculations in the VAX trials failed to increase antibody titers against, CRF01_AE V2, but generated a small response against MN V2 (GMT, 76) in VAX003.

## Conclusion

Antibody responses against V2 were induced by CRF01_A E recombinant proteins as there were no responses induced by the AIDSVAX B/B vaccine regimen. Repeated protein immunization increased the magnitude of responses against recombinant proteins in VAX003 but failed to increase titers against CRF01_AE V2. If antibodies against V2 are protective against HIV-1 acquisition, designing antigens with greater V2 antigenicity would be critical.

